# Development of interoception: the critical role of maternal affective touch in early attachment

**DOI:** 10.3389/fnins.2026.1748486

**Published:** 2026-02-02

**Authors:** Paola Solano Durán, Valeria Isaac

**Affiliations:** 1 Center for Social and Cognitive Neuroscience, School of Psychology, Universidad Adolfo Ibáñez, Santiago, Chile; 2 Oficina de Equidad de Género, Instituto Tecnológico de Costa Rica, Cartago, Costa Rica; 3 Neurosens, Laboratory for Sensory Integration, Santiago, Chile

**Keywords:** C-tactile fibers, early attachment, interoception, interoceptive awareness, maternal affective touch

## Abstract

Despite growing interest in interoceptive functioning, primarily due to its pivotal role in physical and mental health, little is known about its developmental origins and the factors that influence it. Interoception—the perception of one’s internal bodily state—in which bottom-up sensory signals from receptors located in the body are integrated with top-down cognitive interpretations, is crucial for homeostatic and emotional regulation and overall wellbeing. Specific questions remain on how and when this function typically develops. Here, we propose that early attachment relationships, facilitated by maternal affective touch (MAT), provide a critical context in which interoceptive signals are integrated within central nervous system structures and learned to be accurately sensed, perceived, and interpreted later in life. We examine neurobiological mechanisms linking MAT to interoceptive pathways by integrating perspectives from attachment theory and interoception research. We argue that relational experiences and embodied interactions in infancy lay the foundation for lifelong interoceptive awareness and adaptive functioning. These perspectives have implications for developmental models of interoception and for translational approaches to psychopathology characterized by interoceptive and autonomic dysregulation, suggesting that early relational and embodied processes may represent important targets for future research and intervention.

## 1 Introduction

Interoception refers to the nervous system’s capacity to sense, perceive, interpret, and integrate signals originating from within the body (Craig, 2002; Khalsa et al., 2016). This multifaceted process encompasses diverse sensations including visceral activity (e.g., heart rate, respiration, and gastrointestinal sensations), metabolic states (e.g., hunger and thirst), temperature regulation, and pain. Contemporary definitions conceptualize interoception as the phenomenological experience of bodily states—a construct generated by the central nervous system (CNS) through the integration of diverse sensory inputs, regardless of specific receptor pathways involved (Ceunen et al., 2016). This broader conceptualization reflects a theoretical shift from a narrow, receptor-based view to an inclusive framework that positions the CNS as the central architect of interoceptive experience. Interoception operates as a dynamic process wherein bottom-up sensory signals from various body regions are integrated with top-down cognitive and affective interpretations (Seth, 2013). These internal signals, often occurring below conscious awareness, are essential for maintaining physiological homeostasis and supporting higher-order processes including emotional experience, self-awareness, decision-making, and social cognition (Craig, 2009; Critchley and Garfinkel, 2017).

Despite substantial advances in understanding adult interoceptive functioning, limited attention has been devoted to developmental questions: How do interoceptive capacities emerge in early life? What experiential factors shape their maturation and integration? Recent frameworks suggest that interoceptive awareness—defined as metacognitive insight into one’s interoceptive accuracy (Garfinkel et al., 2016)—develops through repeated experiences of bodily states in specific relational and environmental contexts. Yet the mechanisms underlying this development process remain poorly understood. This paper examines a specific developmental pathway: the contribution of early attachment relationships, particularly through maternal affective touch (MAT) as a bodily experience, to the emergence of interoceptive capacities. We propose that MAT provides essential experiential input that calibrates developing interoceptive systems, facilitating the integration of bodily signals with affective and social meaning.

### 1.1 Evolution of the concept of interoception

Sherrington (1906) originally coined the term *interoception* to describe sensory processes conveying information about the body’s internal milieu, primarily through visceral organ sensations. This concept was distinguished from proprioception (sensing body position and movement) and exteroception (sensing external environmental stimuli). However, contemporary understanding of interoception has expanded considerably beyond these initial boundaries (Ceunen et al., 2016). Modern conceptualizations recognize that interoception encompasses not only visceral sensations but also the emotional and cognitive processes contributing to integrated perception of the body’s overall physiological condition (Berntson and Khalsa, 2021). Garfinkel et al. (2016) delineated three key dimensions of interoceptive functioning: (1) interoceptive accuracy (IAcc)—objective performance on behavioral tests of bodily signal detection (e.g., heartbeat counting tasks); (2) interoceptive sensibility (IS)—subjective self-assessment of one’s perceived ability to detect internal signals, typically measured via questionnaires; and (3) interoceptive awareness (IA)—metacognitive insight into the correspondence between one’s interoceptive perceptions and actual accuracy. Interoception provides continuous, moment-by-moment mapping of the body’s internal landscape at both conscious and unconscious levels (Khalsa et al., 2018). It plays crucial roles in fundamental aspects of human experience including sense of self, emotional regulation, empathy, attention, reward processing, and cognitive control (Craig, 2009). Critically, alterations in interoceptive functioning have been implicated in numerous physical and mental health conditions (Khalsa et al., 2016; Nord and Garfinkel, 2022; Solano Durán et al., 2024), underscoring the clinical importance of understanding its developmental trajectory.

### 1.2 Interoceptive afferent pathways and CNS processing

Interoception involves diverse afferent anatomical pathways conveying varied bodily inputs to the CNS. These include cardiovascular parameters, gastrointestinal activity, metabolic states (Tallon-Baudry, 2023), and skin-mediated signals such as thermosensation and affective touch (Crucianelli and Ehrsson, 2023). Accordingly, interoceptors comprise a heterogeneous array of specialized receptors, including chemoreceptors, humoral receptors, mechanoreceptors, and free nerve endings (nociceptors) distributed throughout the body (Berntson and Khalsa, 2021). A useful distinction exists between *early processing* of interoceptive signals—transmitted via homeostatic pathways from periphery to brainstem and thalamus—and *higher-order perception* involving cortical integration and conscious awareness (Gu et al., 2013; Wang et al., 2019). In humans, the insular cortex serves as a primary interoceptive hub, becoming activated when individuals consciously attend to internal bodily states (Craig, 2009). The insula integrates signals from internal and external environments, generating unified representations of bodily state. Three main homeostatic pathways have been characterized:

1. Spinal Homeostatic Pathway: Signals from myelinated Aδ fibers and unmyelinated C-tactile fibers (CTFs) in the skin travel via the spinal cord to brainstem homeostatic regions (nucleus of the solitary tract, parabrachial nucleus), then to thalamic nuclei, ultimately projecting to the posterior dorsal insula—the primary sensory processing area for homeostatic input (Craig, 2010; Ceunen et al., 2016).
2. Cranial Homeostatic Pathway: Cranial nerves (vagus, glossopharyngeal) convey signals to brainstem nuclei [nucleus of the solitary tract (NTS), parabrachial nucleus (PB), periaqueductal gray (PAG)], with subsequent projections to thalamus, hypothalamus, amygdala, anterior cingulate cortex (ACC), and insula (Craig, 2005; Critchley and Harrison, 2013).
3. Humoral Homeostatic Pathway: Non-neural pathway which detects blood-circulating substances such as hormones (secreted by endocrine glands, eg., thyroids, pancreas, adrenal), immune cells and inflammatory mediators (e.g., antibodies, antigens, cytokines), and changes in metabolic signals (glucose, lipids, amino acids, viral particles, waste products among others) via circumventricular organs, blood–brain barrier transport, and microglial pathogen sensing (Berntson and Khalsa, 2021).

While these pathways work in concert to support interoceptive awareness, the spinal homeostatic pathway is particularly relevant to MAT given its CTF afferents, which are specialized for processing the slow, gentle touch characteristic of caregiving interactions.

## 2 Early attachment: theoretical framework

Attachment theory provides an integrative framework for understanding human socio-emotional development, emphasizing that early emotional bonds provide security and enable healthy social interaction (Newman et al., 2015; Bowlby, 1969). Bowlby conceptualized attachment as an innate, homeostatic behavioral system regulating proximity-seeking and contact-maintaining behaviors from infancy. The attachment system is activated during perceived threats or stress, prompting the infant to seek proximity to attachment figures who provide safety, comfort, and affective regulation (Bowlby, 1980).

The quality of early attachment relationships has profound implications for development across multiple domains. Ainsworth (1979) empirical work demonstrated that attachment security depends critically on the quality and consistency of caregiver responsiveness, particularly through physical contact (Feldman et al., 1999; Feldman et al., 2010; Feldman, 2007). Secure attachment develops when caregivers consistently respond to infant signals with appropriate physical and emotional availability. Conversely, inconsistent or insensitive caregiving predicts insecure attachment patterns (anxious or avoidant), which are associated with increased risk for emotional and behavioral difficulties (Bowlby, 1980). Physical contact (e.g., touch) represents a primary modality through which attachment needs are met in early infancy. Securely attached infants actively seek physical/bodily comfort from caregivers, whereas insecurely attached infants may show ambivalence or avoidance of such physical contact (Ainsworth et al., 2015). The fundamental importance of tactile contact for attachment has been demonstrated in both human and animal research (Yoshida and Funato, 2021).

### 2.1 Embodied nature of early attachment

Recent theoretical developments emphasize the fundamentally embodied nature of early attachment relationships. Montirosso and McGlone (2020) propose that maternal interoceptive sensitivity—the mother’s capacity to accurately perceive and respond to her own bodily states—underlies her ability to attune effectively to infant bodily signals. From this perspective, the infant’s emerging sense of bodily self is not solely an individual achievement but arises through shared, inter-corporeal processes in which the caregiver plays a constitutive role (Ciaunica and Crucianelli, 2019). Marshall and Meltzoff (2015) similarly emphasize that bodily perception develops through relational and emotional exchanges rather than purely maturational processes. Research indicates that development of the bodily self in infancy depends not only on brain–body interactions but fundamentally on body-to-body experiences—embodied interactions that lay foundations for the minimal self, a crucial precursor to self-other differentiation (Fotopoulou and Tsakiris, 2017; Ciaunica and Crucianelli, 2019; Hornor, 2019; van Rosmalen et al., 2014). This theoretical framework suggests that interoceptive development may be inherently relational, emerging from repeated embodied exchanges between infant and caregiver, with MAT as a potential mechanism for these exchanges.

## 3 Maternal affective touch: characteristics and functions

**Figure 1:**
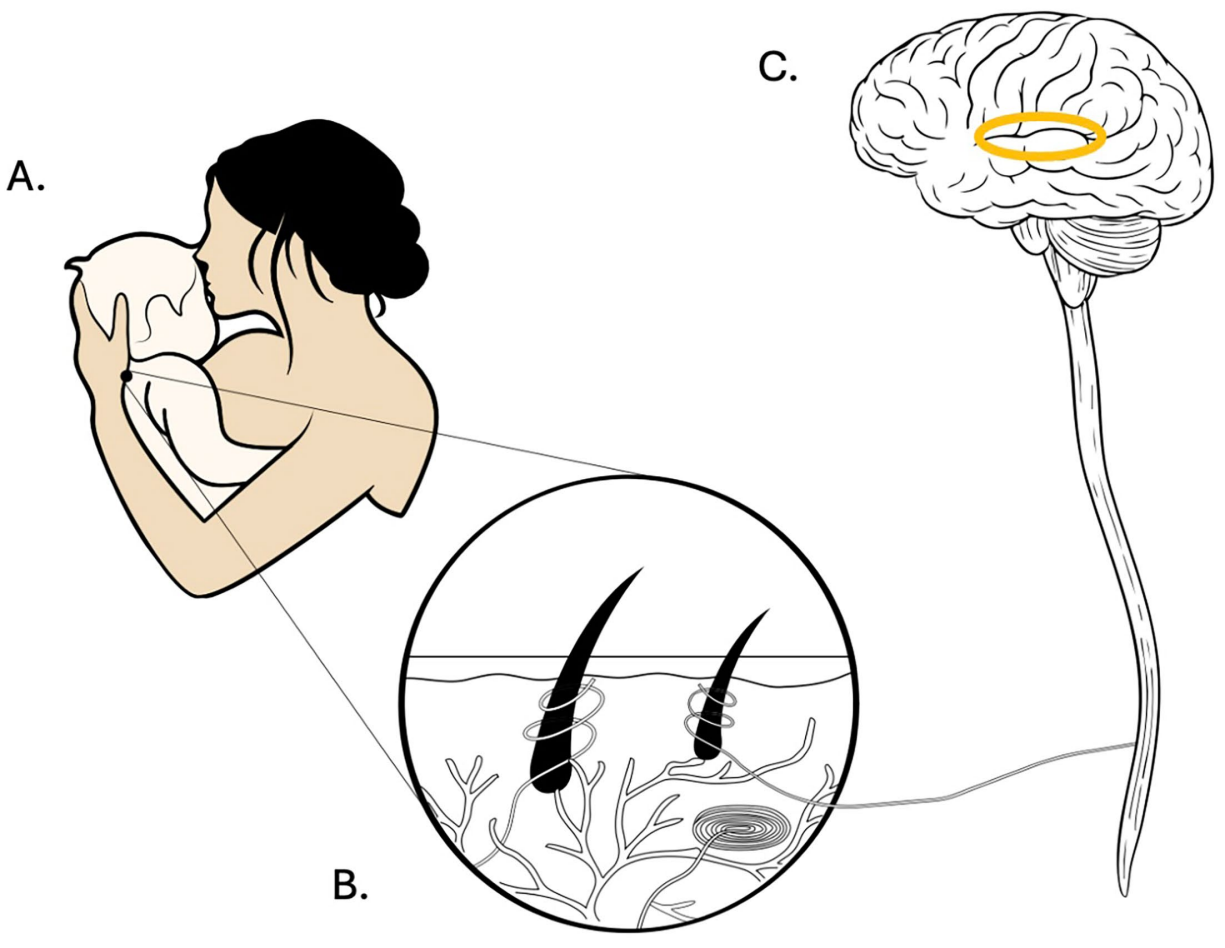
Illustration of the neural pathway that follows repeated experiences of MAT during infancy. **(A)** Mother and child. Mother’s caregiving touch (MAT) characterized by slow stroking movement and affective intentionality and conducive to emotional bonding. **(B)** Hairy skin showing CTFs. Activation of specialized peripheral tactile receptors (CTFs) tuned to socially relevant tactile stimulation such as MAT. **(C)** Insular cortex. CTF’s travel to the CNS via the homeostatic pathways integrating tactile input in the insular cortex and associated regions, providing foundations for emotional regulation and social cognition.

From birth, infants engage in continuous body-to-body interactions with caregivers through routine care activities including feeding, diaper changes, and social play. These embodied interactions involve multimodal coordination of behaviors and physiological states, contributing to interpersonal rhythmic cycles of co-regulation (Montirosso and McGlone, 2020). This bidirectional regulatory process—often termed mutual regulation (co-regulation) or attunement (Tronick, 1989; DiCorcia and Tronick, 2011)—is facilitated by face-to-face interaction, physical proximity, and tactile contact such as MAT (Figure 1A). MAT refers to caregiving touch (slow light pressure and gentle stroking) characterized by affective intentionality and conducive to emotional bonding. This form of tactile interaction has been consistently associated with secure attachment in healthy infants, beyond effects attributable to general maternal sensitivity (Weiss et al., 2000; Botero et al., 2020). MAT leverages infants’ natural preferences for maternal sensory cues including scent, voice, and tactile qualities (Vaglio, 2009).

### 3.1 Developmental importance of MAT

The tactile system develops remarkably early, with functional receptors present from approximately 4–7 weeks of gestation (Bremner and Spence, 2017). Touch provides crucial information about both physical and social environments, profoundly influencing emotional experience and social interaction. Caregivers employ diverse tactile modalities with distinct communicative purposes (Jean and Stack, 2012). These include slow, gentle strokes conveying affection; playful touches such as tickling; supportive touches for behavioral regulation; and directive touches guiding attention. Each touch modality functions as a specific communicative signal, paralleling the specificity of facial expressions or vocal prosody in conveying meaning to the child (Tronick, 1995). Research demonstrates that touch in close relationships enhances attachment security and contributes to relational and psychological well-being (Jakubiak and Feeney, 2016, 2017). Importantly, individual differences in attachment style predict attitudes toward and use of affective touch: individuals with avoidant attachment show more negative attitudes toward social touch, whereas those with anxious attachment more frequently seek and initiate affective touch (Ekeberg, 2016). These patterns appear stable across development, with childhood and adolescent touch experiences strongly predicting adult attachment styles and social touch behaviors (Beltrán et al., 2020).

Higher levels of MAT during the early postnatal months are associated with positive cognitive and neurobehavioral outcomes (Botero et al., 2020). Such interactions facilitate bio-behavioral synchrony—coordination of physiological, neuroendocrine, and behavioral responses essential for social development (Feldman, 2012; Feldman et al., 2014; Atzil and Gendron, 2017). Furthermore, through associations with oxytocin and other neuromodulators, MAT may modulate precision during social allostasis, adjusting homeostatic setpoints in response to social context and long-term developmental needs (Krahé et al., 2013; von Mohr and Fotopoulou, 2018; Fotopoulou et al., 2022). Recent research indicates that MAT, mediated by C-tactile fiber activation, appears to promote an infant’s somatosensory system development, autonomic regulation, immune function, reward processing and learning (Carozza and Leong, 2021) in addition to enhancing caregiver-infant bonding, which reduces stress responses and supports neurodevelopment (La Rosa et al., 2024). Notwithstanding, clinical applications of structured touch interventions (e.g., infant massage, kangaroo care) demonstrate benefits for preterm infants including enhanced weight gain, pain management, shortened hospitalizations, neurodevelopmental support, and reduced parental stress (Field, 2019).

The affective touch system is functional from early postnatal life, capable of distinguishing affective from non-affective touch stimulation (Jönsson et al., 2018). This system plays a fundamental role in social bonding, emotional communication, and—critically for the present argument—interoceptive development (Liljencrantz and Olausson, 2014; McGlone et al., 2014).

## 4 Neurobiological mechanisms: maternal affective touch and interoceptive pathways

### 4.1 C-tactile fibers: peripheral encoding of MAT

Neurophysiological research has identified parallel neural systems for processing different aspects of touch: one for discriminative touch and another for affective touch (McGlone et al., 2014). The discriminative touch system, mediated by fast-conducting myelinated afferents (Aβ fibers), processes tactile properties including location, intensity, and texture. In contrast, the affective touch system, mediated by slow-conducting unmyelinated C-tactile fibers (CTFs), specifically processes the hedonic and social-affective qualities of touch.

At the peripheral level, positively valenced affective tactile sensations are encoded by specialized CTFs. These unmyelinated fibers are present exclusively in hairy skin, absent from glabrous (non-hairy) surfaces such as palms and soles. Their lack of myelination accounts for slow conduction velocity (0.6–1.3 m/s) and resulting delay between stimulus onset and cortical processing (Ackerley et al., 2014; Figure 1B). Microneurography studies demonstrate that CTFs respond selectively to specific tactile parameters: gentle stroking at slow velocities (1–10 cm/s) delivered at skin-like temperature (~32 °C). CTF activation correlates closely with subjective ratings of pleasantness (Johansson and Vallbo, 1979; Löken et al., 2009; Ackerley et al., 2014). This tuning profile aligns closely with the defining features of typical caregiving touch, understood as routine, affectively meaningful, and contingently responsive tactile interactions that support caregiver–infant co-regulation (Feldman, 2012; Montirosso and McGlone, 2020), suggesting an evolutionary adaptation for processing socially relevant tactile signals.

### 4.2 Central processing of MAT

MAT operates through three interconnected neurobiological mechanisms (Fotopoulou et al., 2022): (1) attachment regulation, satisfying innate predictions about social proximity via hedonic, dopaminergic, and opioidergic pathways (Panksepp, 2004); (2) homeostatic co-regulation, wherein caregiving touch (e.g., warming the infant) directly modulates physiological states through autonomic and endocrine pathways; and (3) allostatic modulation, whereby gentle affective touch enhances salience and epistemic value of experiences in specific contexts via oxytocin release and neuromodulatory circuits (Fotopoulou and Tsakiris, 2017).

Peripheral CTF signals are transmitted via the spinal homeostatic pathway to the posterior insular cortex, a primary hub for interoceptive processing. From the insula, information projects to the orbitofrontal cortex and anterior cingulate cortex (ACC)—regions involved in affective evaluation, reward processing, and social cognition (Björnsdotter et al., 2010; Rolls, 2004; Craig, 2009). The insular cortex serves as the principal interoceptive integration center, combining signals from internal organs with exteroceptive sensory inputs to generate unified bodily representations (Tsakiris, 2017; Figure 1C).

Developmental neuroimaging studies demonstrate insular functionality from early postnatal life: 2-month-old infants show greater insular activation to slow stroking (optimal for CTF stimulation) compared to faster stroking velocities (Fairhurst et al., 2014; Jönsson et al., 2018; Tuulari et al., 2019). Longitudinal research provides further evidence linking early tactile experience to interoceptive neural development. Children who experienced higher levels of maternal touch show increased structural and functional connectivity in right-hemisphere insula and ACC (Brauer et al., 2016). MAT also supports experience-dependent plasticity in somatosensory cortex: gentle stroking in infancy preferentially activates primary somatosensory cortex and posterior insula, regions critical for encoding location, intensity, and affective valence of touch (Carozza and Leong, 2021). These early tactile experiences thus contribute directly to maturation of interoceptive circuits underlying higher-order cognitive and socio-emotional functions.

### 4.3 MAT and autonomic regulation

As a modality of interoceptive signaling, MAT influences parasympathetic tone—vital for maintaining physiological homeostasis and signaling safety to the organism (Krahé et al., 2016; Püschel et al., 2022). Affective touch communicates bidirectional information: bottom-up signals about one’s own bodily state and top-down contextual information about social interaction (Montirosso and McGlone, 2020). Touch conveys information about available social resources and modulates physiological responses including pain perception based on expectations of social support (Krahé et al., 2016).

Critically, infants do not process interpersonal touch merely as mechanosensory stimulation; their responses depend on the source and relational context of touch. Aguirre et al. (2019) demonstrated that infants show greater heart rate deceleration—indicative of parasympathetic activation and attentional engagement—when stroked by their mother compared to a stranger. Importantly, this effect occurred specifically for touch velocities optimal for CTF activation, suggesting that the affective touch system is already socially tuned in early infancy.

## 5 Interoceptive development: future considerations

Despite challenges in measuring interoceptive functioning in preverbal populations, emerging evidence indicates that interoceptive capacities are present from early development, with abilities varying across growth stages in parallel with cognitive maturation and psychopathology risk periods (Murphy et al., 2017). Most pediatric interoception research has adapted adult methodologies, relying heavily on heartbeat perception tasks (Koch and Pollatos, 2014) and self-report measures such as the Multidimensional Assessment of Interoceptive Awareness (MAIA; Jones et al., 2021). However, implicit interoceptive functioning—automatic processing of bodily signals without conscious awareness—appears to emerge considerably earlier than explicit capacities. Evidence suggests implicit interoception may be present from birth or even prenatally (Zieber et al., 2014; Klabunde, 2019). Fairhurst et al. (2014) observed heart rate deceleration in 9-month-old infants in response to caregiver stroking at medium velocity (~3 cm/s)—the velocity optimal for CTF activation. Maister et al. (2017) provided evidence for implicit interoceptive sensitivity in 5-month-old infants, who showed longer visual fixations on characters moving asynchronously with their heartbeat, indicating detection of interoceptive-visual mismatches. These findings suggest that even preverbal infants possess functional interoceptive systems capable of detecting internal bodily signals and integrating them with exteroceptive information. The developmental trajectory from implicit detection to explicit awareness and metacognitive insight (IA) needs further research (Quattrocki and Friston, 2014).

Since interoception involves perceiving internal physiological states, its development requires constructing body awareness through accurate interpretation of incoming signals. Evidence shows that early embodied interactions with both physical and social environments are crucial for the healthy development of body awareness (Parma et al., 2024). Physical environment/object interactions occur primarily through tactile exploration: infants naturally grasp, touch, and mouth objects within reach. Evidence indicates this exploratory behavior is essential not only for motor development but also for higher cognitive functions, including conceptual development and mental representations (Barsalou, 2010; Novak and Schwan, 2021). However, during critical early developmental periods, neural pathways are shaped not merely by physical object interaction but profoundly by social proximity exchanges with caregivers (Gagliardi, 2023). Here, social interaction represents infants’ most important and preferred form of engagement (Ilyka et al., 2021), with MAT being a primary medium for interoceptive maturation during these interactions.

This perspective positions interoception not as a purely individual capacity but as fundamentally relational, emerging from co-regulatory processes between infant and caregiver (Fonagy and Campbell, 2017). Supporting this view, children raised in institutional settings—characterized by inconsistent caregiving and limited physical contact—frequently exhibit sensory dysregulation, including heightened sensory reactivity, increased prevalence of sensory processing disorders, and varying degrees of touch aversion (Dozier et al., 2002; Wilbarger et al., 2010). Disruptions in interoceptive development as early as infancy, such as reduced tactile input or inconsistent co-regulation, may contribute to later deficits in emotional regulation and decision-making (Sugawara et al., 2020; Murphy et al., 2017).

Research evidence on alternative explanations or confounding factors that may influence the development and maturation of interoception largely centers on the quality of early-life environments and the nature of caregiver interactions, aside from MAT in early attachment. Beyond the physical proximity and interaction, caregivers contribute to the development of IA by accurately interpreting the infant’s internal distress cues (e.g., crying due to hunger or discomfort) and responding promptly and appropriately (Filippetti, 2021). The child then learns to associate their internal sensation with a specific need and subsequent relief, which helps build a reliable internal reference system, forming the basis for self-regulation and emotional awareness. Acknowledging and validating the child’s expressions and emotions, and verbalizing their children’s bodily sensations, is critical for fostering adaptive emotional development and, by extension, healthy IA (Addabbo and Milani, 2025). Conversely, adverse experiences during early developmental periods can significantly disrupt interoceptive maturation. When external caregiver regulation is absent or inconsistent, children may become overwhelmed by unintegrated states of distress. This can lead to the suppression, avoidance, or disregard of bodily feelings as a survival coping mechanism, resulting in lower interoceptive accuracy and awareness (Oldroyd et al., 2019). Even more so, early exposure to acute stress, trauma, and abuse can permanently alter the brain structures critical for interoception, predisposing individuals to mental and physical health vulnerabilities later in life (Schaan et al., 2019; Petrenchik, 2025). Also, prenatal influences, including maternal trauma and stress, inflammation, endocrine disruption, or substance exposure, can alter the development of brain structures and pathways implicated in interoception before postnatal caregiving even begins (Lubrano et al., 2024).

Other lifestyle factors possibly influencing the development of interoception from early life may be related to natural environment-based interactions. Engaging in movement and play in natural environments (e.g., grass, forests, beaches) may promote IA by enhancing sensory experiences and providing vital tactile, proprioceptive, and vestibular input (Summers et al., 2019). Another factor that has shown a consistent link with interoception is the gut-brain axis, showing that alterations in gut microbiota composition could modulate interoceptive processing through immune signaling and neurotransmitter production (Kano and Fukudo, 2025). Graf et al. (2025) revealed a connection between the early gut microbiome and the structure of the insula in neonates, highlighting the gut-brain axis’s potential implications for socio-emotional function and IA. These findings suggest that the gut microbiome in infants acts as a critical environmental factor influencing the development of interoceptive neural circuitry and laying the groundwork for processing bodily sensations and emotions.

Further research integrating these multiple dimensions—from maternal interactions and environmental engagement to gut microbiome composition—will be essential for understanding the holistic development of interoceptive abilities in early life.

## 6 Conclusion

The developmental significance of interoception goes well beyond the maintenance of internal physiological stability; it is fundamental to the emergence of bodily self-awareness, emotional regulation, and socio-affective functioning (Crucianelli et al., 2018). The evidence reviewed supports a developmental perspective wherein early attachment relationships, mediated particularly by MAT, provide essential experiential scaffolding for interoceptive system maturation and the construction of the embodied self, with lasting implications for mental health and socio-emotional competencies.

Advancing our understanding of the relational and embodied origins of interoception may inform effective preventive and therapeutic interventions aimed at fostering emotional and social well-being from infancy onward. Early attachment through MAT offers a compelling integrative framework for understanding not only the roots of socioemotional development but also the early foundations of interoception. This framework suggests that interoceptive development is not purely maturational but fundamentally shaped by relational experiences through the form of MAT. The quality of early attachment—reflected in consistency, sensitivity, and physical affection of caregiving through MAT—may establish templates for how bodily signals are interpreted and integrated with emotional and social meaning throughout life, resulting in more accurate and adaptive IA. Elucidating how MAT interacts with epigenetic, prenatal, and postnatal factors to shape interoceptive circuits will be crucial for refining developmental models and informing early interventions.

## Data Availability

The original contributions presented in the study are included in the article/supplementary material, further inquiries can be directed to the corresponding authors.
